# Epidemiological analysis of the distribution of cystic and alveolar echinococcosis in Osh Oblast in the Kyrgyz Republic, 2000–2013

**DOI:** 10.1017/S0022149X15000565

**Published:** 2015-11

**Authors:** K.M. Raimkylov, O.T. Kuttubaev, V.S. Toigombaeva

**Affiliations:** Kyrgyz State Medical Academy, 92, Akhunbaeva Str, Bishkek, Kyrgyzstan

## Abstract

Alveolar and cystic echinococcosis are highly endemic in the Kyrgyz Republic. This report documents the numbers of recorded cases of these two diseases that have been reported in the past 14 years. The number of cases of echinococcosis has increased from approximately 550 to 1044 cases in 2013. This is an increase in incidence from 11.3 to 18.3 cases per 100,000 annually. In 2000 no cases of alveolar echinococcosis (AE) were reported in the Kyrgyz Republic. During this period the disease has emerged, with 148 cases reported in 2013 (2.6 cases per 100,000). Osh Oblast is a highly endemic focus for AE, with 60 cases reported in 2013 (6.0 per 100,000). The Alay Valley in the south of Osh Oblast reported the majority of AE cases for this region. In this valley, in 2013, 42 cases of AE were reported, which is a local incidence of 58 per 100,000.

## Introduction

Parasitic diseases remain a serious health burden on human populations. Of approximately 1415 known human pathogens, 287 are helminths species and a further 66 species are protozoan pathogens (Taylor *et al.*, 2001). Human cystic echinococcosis (CE) is caused by the larval stage of *Echinococcus granulosus* and human alveolar echinococcosis (AE) is caused by the larval stage of *E. multilocularis* (Torgerson & Budke, 2003). Both of these parasites are endemic in Kyrgyzstan (Torgerson *et al.*, 2003; Usubalieva *et al.*, 2013) and both cause serious morbidity and disease burden (Budke *et al.*, 2006; Torgerson *et al.*, 2010).

AE is one of the most dangerous diseases for humans and is a typical focal zoonotic disease. It is characterized by a severe but slowly progressing infiltrative disease, with the initial lesion usually in the liver followed by metastases to the brain, lungs and other organs. In the absence of intensive medical treatment, the outcome is usually fatal (Torgerson *et al.*, 2008). The definitive hosts of *E. multilocularis* include foxes, dogs and wolves. Natural intermediate hosts include many species of small mammals, whereas humans are an aberrant intermediate host. Livestock are not usually affected by this parasite and hence the epidemiology is very different to that of *E. granulosus*. Epidemiological and epizootiological studies of cystic and alveolar echinococcosis have been conducted by Bolokh (1965), Abdyjaparov (1997), Torgerson *et al.* (2003), Abdyjaparov & Kuttubaev (2004), Karaeva (2004), Raimkulov *et al.* (2008) and Ziadinov *et al.* (2008).

Kyrgyzstan is a well-known hyperendemic region for CE and the reported incidence has doubled over the past 10 years compared to the previous 20 years. However, the country is also highly endemic for AE, with marked increases in some districts of northern Kyrgyzstan (Raimkulov *et al.* 2008) as well as in southern districts, particularly Osh Oblast. The numbers of cases of AE notified indicate an emerging epidemic of human disease. Until the end of the 20th century, the disease appeared to be rare and sporadic. In recent years substantial numbers of cases have been treated (Usubalieva *et al.*, 2013).

The true incidence of the disease, together with the epidemiology of infection and disease trends, has not been studied in Osh Oblast. The purpose of this study was to further characterize the epidemiology of cystic and alveolar echinococcosis in endemic areas of the Kyrgyz Republic, and so make a contribution to optimizing the control of these diseases. To achieve this for the Kyrgyz Republic in general, and Osh Oblast in particular, we have conducted a retrospective analysis of the statistical disease reports, to establish the likely true incidence of CE and AE from records in city, regional and district hospitals. We have used these data to map the distribution of the disease in Osh Oblast.

## Materials and methods

CE and AE incidences were estimated from reports of medical institutes and data from the State Department of Sanitary and Epidemiological Surveillance of the Kyrgyz Ministry of Health and the Kara-Suu district centre of disease prevention and sanitary and epidemiological surveillance. Case definitions were those that were confirmed as CE or AE by morphological and histological analysis of lesions following resection. Incidence per 100,000 was calculated from numbers of cases reported and the population size of districts. Confidence intervals were based on exact Poisson confidence intervals of the observed counts and the population size of the districts from which the cases were reported.

## Results and discussion

**Fig. 1:**
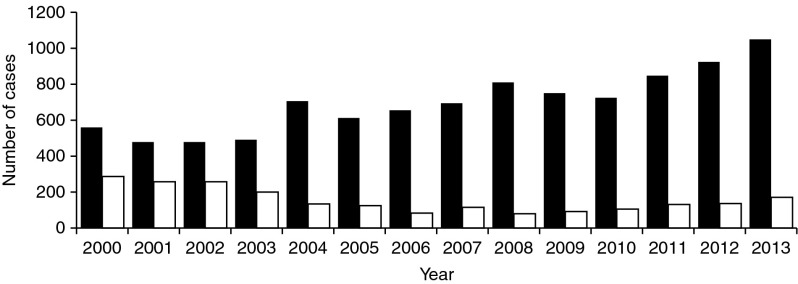
Echinococcosis in the Kyrygz Republic between 2000 and 2013. Official statistics report the total number of cases (black bars) (including men, women and children) and the number in children aged up to 14 years (white bars).

There has been a substantial increase in CE and AE in animals and humans since the collapse of the Soviet Union. The reported data show that in recent years in Kyrgyzstan the number of cases of CE or AE has been about 800–1000 per year. This is accompanied by significant social and economic losses and a deterioration in general health. CE and AE are recorded throughout the country, especially where livestock populations are high. The districts with the highest numbers of cases of CE and AE appeared to be Osh, Naryn and Jalal-Abad. The total numbers of cases of echinococcosis reported between 2000 and 2013 are shown in fig. 1. Although children accounted for up to one-third of cases in 2000–2003, this proportion has now become much smaller.

**Fig. 2:**
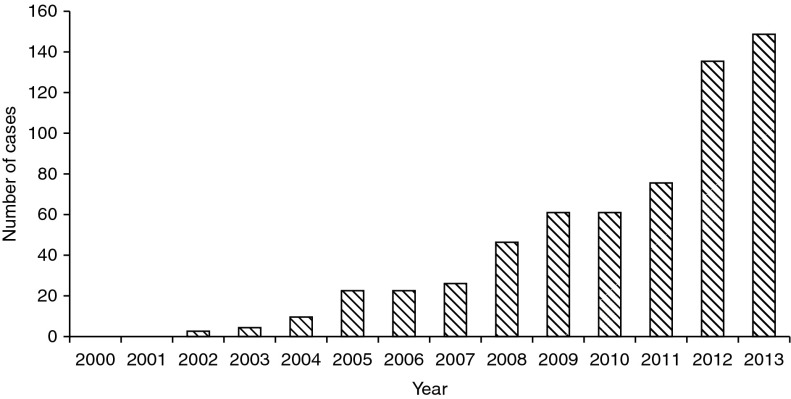
The number of cases of AE reported in Kyrgyzstan between 2000 and 2013.

In the Kyrgyz Republic in 2007 there were 695 cases of echinococcosis, of which 26 were AE. By 2013 this had increased to 1049 cases of which 148 were AE (fig. 2). The number of cases of AE reported has increased from between 0 and 9 cases per year between 2000 and 2004, to 148 cases reported in 2013.

**Fig. 3:**
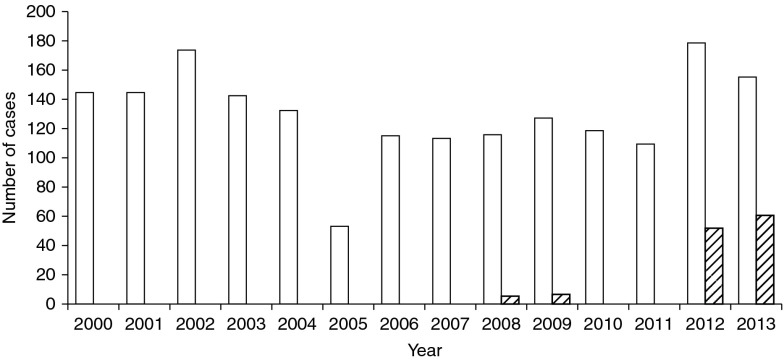
The numbers of CE (white bars) and AE cases (shaded bars) notified in Osh Oblast between 2000 and 2013.

However, the official statistics appear to underestimate the true numbers of cases. Analysis of hospital records suggests that in 2001 there were 12% more cases of echinococcosis than officially stated. The Ministry of Health has recorded the place of origin of all cases of echinococcosis. The total numbers of cases of echinococcosis reported in Osh Oblast between 2000 and 2013 were 1855 cases of CE and 122 cases of AE. All the cases of AE have been notified since 2008 (fig. 3). One of the aggravating factors is the frequent recurrence of the disease, which leads to complications and death. Of 109 cases in Osh Oblast in 2011, 9 were reported as recurrent cases.

The problem with AE is that it runs a chronic course of infection and is usually diagnosed at a late stage. Consequently, surgical treatment is often undertaken when the disease has reached an advanced stage, there are frequent complications and, consequently, the disease is often fatal. A focus of AE has emerged in the Alay district of Osh Oblast. These data confirm the urgent need to study the transmission of the disease in this district and to implement appropriate control measures. Table 1 gives the number of cases reported by district within Osh Oblast and the incidence per 100,000 in 2013.

**Table 1:** The total numbers of cases and the annual incidence of CE and AE per 100,000 reported by district in Osh Oblast in 2013 (CIs, 95% confidence intervals).

District	CE	Incidence (CIs)	AE	Incidence
Aravan	0	0 (0–3.6)	0	0 (0–3.6)
Uzgen	34	17 (11.5–23.7)	0	0 (0–1.8)
Kara-Kulija	9	10 (4.7–19.5)	3	3.4 (0.7–10.0)
Nookat	36	17 (11.4–22.1)	2	0.9 (0.1–3.1)
Kara-Suu	65	20 (15.3–25.2)	5	1.6 (0.5–3.5)
Alay	11	15 (7.6–27.3)	42	58 (42–79)
Chon-Alay	0	0 (0–15.8)	8	34 (14–66)
Total	155	16 (13.2–18.1)	60	6.0 (4.5–7.7)

Statistical analysis using a Poisson model indicates that the distribution of AE and CE varies according to district. Alay and Chon-Alay districts have higher numbers of AE cases than the remainder of Osh Oblast (*P* <  0.001), although there is no significant difference between the two districts. Uzgen district, with an upper confidence value of 1.8 cases per 100,000 may also have a lower incidence than Kara-Kulija district. For CE the Alay, Uzgen, Kara-Kulija, Nookat and Kara-Suu districts have a significantly higher incidence than Aravan and Chon-Alay districts (table 1).

These data suggest that there is a focus of high incidence of AE in Alay and Chon-Alay districts in Osh Oblast. The annual incidence confined to these districts was 58 and 34 cases per 100,000, respectively. Although highly localized, such high incidences of AE have rarely been reported. Even for the much larger region of Osh Oblast, an incidence of 6 cases per 100,000 is very high. Likewise, for CE the incidence of 16 cases per 100,000 for Osh Oblast is also remarkably high. The large numbers of CE cases now being reported in the Kyrgyz Republic are linked to the dissolution of the Soviet Union, changes in farming practices, closure of meat-processing plants and economic collapse. This has been described in detail elsewhere (Torgerson, 2013). Likewise, the reasons for the epidemic of AE in Kyrgyzstan have been speculated to be linked to the collapse of the Soviet Union, the subsequent expansion of the dog population and colonization of dogs with *E. multilocularis* (Torgerson, 2013). A high prevalence of infection of dogs with *E. multilocularis* has been reported in Naryn Oblast (Ziadinov *et al.*, 2008), together with a high prevalence in foxes (Ziadinov *et al.*, 2010). In the Alay valley, coproantigen studies have revealed a high prevalence of infection with *Echinococcus* spp. in dogs (Mastin *et al.*, 2015). In this district there is also widespread contamination of the environment with dog faeces, with mean canine faecal densities ranging from 0.22 faeces per 100 m^2^ to 1.2 faeces per 100 m^2^. Such faeces have been proven by polymerase chain reaction (PCR) to contain eggs of *E. multilocularis*, *E. granulosus* or both (Van Kesteren *et al.*, 2013).

These data confirm the alarming epidemic reported by Usubalieva *et al.* (2013), but update this and demonstrate that the numbers of AE cases reported in Kyrgyzstan have doubled between 2011 and 2013. Furthermore, there is a large focus of the disease in the Alay valley, although cases are being reported from every region of the country.

## Conflict of interest

None.

## References

[ref1] Abdyjaparov T.A. (1997) Role of rodents in the formation of natural foci of alveolar echinococcosis in the territory of high altitude pastures in the Kyrygz republic. Summary of the Dissertation for the Candidate of Biological Sciences, Kyrgyz State Medical Academy, Bishkek (in Russian).

[ref2] Abdyjaparov T.A. & Kuttubaev O.T. (2004) Alveolar echinococcosis in rodents of mountainous pastures of Kyrgyzstan. pp. 253–262 *in* Torgerson, P.R. & Shaikenov, B. *(Eds) Echinococcosis in central Asia: problems and solutions*. Dauir Almaty ISBN 9965-517-92-4.

[ref3] Bolokh Y.A. (1965) Human cystic and alveolar echinococcosis. 180 pp. Frunze, Academy of Sciences of the Kyrgyz SSR (in Russian).

[ref4] Budke C.M., Deplazes P. & Torgerson P.R. (2006) Global socioeconomic impact of cystic echinococcosis. Emerging Infectious Diseases 12, 296–303. 16494758 10.3201/eid1202.050499 PMC3373106

[ref5] Karaeva R.R. (2004) Contemporary optimisation of epidemiological surveillance in the Kyrgyz Republic Dissertation for Degree of Candidate of Biological Sciences, Kyrgyz State Medical Academy, Bishkek (in Russian).

[ref16] Mastin J., van Kesteren F., Torgerson P.R., Ziadinov I., Mutunova B., Rogan M.T., Tursonov T. & Craig P.S. (2015) Risk factors for *Echinococcus* coproantigen positivity in dogs from the Alay Valley, Kyrgyzstan. Journal of Helminthology, in press. 10.1017/S0022149X15000590 PMC4700908 26442706

[ref17] Raimkulov K.M., Abdykerimov K.K., Karayeva R.R., Abdyzhaparov T.A., Kuttubayev O.T. & Kozlov S.S. (2008) Natural and synanthropic foci of *Echinococcus alveolaris* infection (alveococcosis) in the northern areas of Kyrgyzstan. Medical Parasitology and Parasitic Diseases 1, 22–25 (in Russian). 18365469

[ref6] Taylor L.H., Latham S.M. & Woolhouse M.E. (2001) Risk factors for human disease emergence. Philosophical Transactions of the Royal Society B: Biological Sciences 356, 983–989. 10.1098/rstb.2001.0888 PMC1088493 11516376

[ref19] Torgerson P.R. (2013) The emergence of echinococcosis in central Asia. Parasitology 140, 1667–1673. 23659353 10.1017/S0031182013000516

[ref7] Torgerson P.R. & Budke C.M. (2003) Echinococcosis – an international public health challenge. Research in Veterinary Science 74, 191–202. 12726737 10.1016/s0034-5288(03)00006-7

[ref8] Torgerson P.R., Karaeva R.R., Corkeri N., Abdyjaparov T.A., Kuttubaev O.T. & Shaikenov B.S. (2003) Human cystic echinococcosis in Kyrgyzstan: an epidemiological study. Acta Tropica 85, 51–61. 12505183 10.1016/s0001-706x(02)00257-7

[ref9] Torgerson P.R., Schweiger A., Deplazes P., Pohar M., Reichen J., Ammann R.W., Tarr P.E., Halkik N. & Müllhaupt B. (2008) Alveolar echinococcosis: from a deadly disease to a well-controlled infection. Relative survival and economic analysis in Switzerland over the last 35 years. Journal of Hepatology 49, 72–77. 18485517 10.1016/j.jhep.2008.03.023

[ref10] Torgerson P.R., Keller K., Magnotta M. & Ragland N. (2010) The global burden of alveolar echinococcosis. PLoS Neglected Tropical Diseases e722 doi:10.1371/journal.pntd.0000722. 10.1371/journal.pntd.0000722 PMC2889826 20582310

[ref11] Usubalieva J., Minbaeva G., Ziadinov I., Deplazes P. & Torgerson P.R. (2013) Human alveolar echinococcosis in Kyrgyzstan. Emerging Infectious Diseases 19, 1095–1097. 23763935 10.3201/eid1907.121405 PMC3713972

[ref13] Van Kesteren F., Mastin A., Mytynova B., Ziadinov I., Boufana B., Torgerson P.R., Rogan M.T. & Craig P.S. (2013) Dog ownership, dog behaviour and transmission of *Echinococcus* spp. in the Alay Valley, southern Kyrgyzstan. Parasitology 140, 1674–1684. 23985326 10.1017/S0031182013001182 PMC3806042

[ref12] Ziadinov I., Mathis A., Trachsel D., Rysmukhambetova A., Abdyjaparov T.A., Kuttubaev O.T., Deplazes P. & Torgerson P.R. (2008) Canine echinococcosis in Kyrgyzstan: using prevalence data adjusted for measurement error to develop transmission dynamics models. International Journal for Parasitology 38, 1179–1190. 18371969 10.1016/j.ijpara.2008.01.009 PMC2527539

[ref14] Ziadinov I., Deplazes P., Mathis A., Mutunova B., Abdykerimov K., Nurgaziev R. & Torgerson P.R (2010) Frequency distribution of *Echinococcus multilocularis* and other helminths of foxes in Kyrgyzstan. Veterinary Parasitology 171, 286–292. 20434845 10.1016/j.vetpar.2010.04.006 PMC2903646

